# The comparison of health‐related quality of life and patient satisfaction between single‐incision and multiport laparoscopic colectomy for cancer: A sub‐study of a randomized, prospective clinical trial

**DOI:** 10.1002/ags3.12378

**Published:** 2020-07-23

**Authors:** Hiroki Ohya, Jun Watanabe, Yusuke Suwa, Hirokazu Suwa, Mayumi Ozawa, Atsushi Ishibe, Shoichi Fujii, Kazumi Kubota, Chikara Kunisaki, Itaru Endo

**Affiliations:** ^1^ Department of Gastroenterological Surgery Graduate School of Medicine Yokohama City University Yokohama Japan; ^2^ Department of Surgery Gastroenterological Center Yokohama City University Medical Center Yokohama Japan; ^3^ Department of Surgery Yokosuka Kyosai Hospital Yokosuka Japan; ^4^ Department of Biostatistics Yokohama City University School of Medicine Yokohama Japan

**Keywords:** colorectal surgery, health‐related quality of life, laparoscopic surgery, patient satisfaction, single‐incision

## Abstract

**Aim:**

The present study clarified the effect on the health‐related quality of life and patient satisfaction of single‐incision laparoscopic colectomy compared with multiport laparoscopic colectomy for colorectal cancer.

**Methods:**

We conducted a multicenter, randomized, control trial comparing single‐incision and multiport laparoscopic colectomy for colon cancer. We performed a pre‐planned secondary analysis of health‐related quality of life and patient satisfaction data of 200 patients. Health‐related quality of life was evaluated using the Japanese 36‐item Short Form Health Survey (SF‐36) version 2.0 before surgery and at 1 month after surgery. Patient satisfaction was compared using seven questionnaires at 1 month after surgery.

**Results:**

One hundred patients were assigned to each group. After excluding 18 patients (9.0%) who did not complete the SF‐36, 182 patients (91.0%) were included in the analysis (92 cases of single‐incision laparoscopic colectomy and 90 cases of multiport laparoscopic colectomy). The SF‐36 scores at 1 month after surgery were not significantly different between the two arms. The role physical, bodily pain, vitality and physical component summary were significantly lower at 1 month after surgery than before in both groups. However, the role emotional was significantly lower after surgery than before only in the single‐incision laparoscopic colectomy group. In terms of patient satisfaction at 1 month after surgery, there were no significant differences in any of the seven items on the questionnaire.

**Conclusions:**

Single‐incision laparoscopic colectomy was similar to multiport laparoscopic colectomy in terms of health‐related quality of life and patient satisfaction. However, single‐incision laparoscopic colectomy may be inferior than multiport laparoscopic colectomy in terms of the role emotional.

## Graphical abstract

We demonstrated in this sub‐analysis of a multicenter, randomized controlled trial comparing single‐incision laparoscopic colectomy (SILC) and multiport laparoscopic colectomy (MPLC) for colon cancer patients that SILC was similar to MPLC in terms of health‐related quality of life and satisfaction. However, SILC may be inferior in terms of the role emotional.

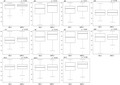

## 1 INTRODUCTION

Colorectal cancer (CRC) is the third‐most commonly diagnosed cancer in the world and the second‐most common cause of cancer death after lung cancer, and its incidence among causes of death is increasing annually.^1^, ^2^, ^3^ Surgery is the only radical treatment for advanced CRC, and laparoscopic surgery has been widely accepted as a standard surgical procedure for CRC.^4^, ^5^

In recent years, there have been an increasing number of reports of single‐incision laparoscopic colectomy (SILC).^6^, ^7^, ^8^, ^9^, ^10^, ^11^, ^12^, ^13^, ^14^ Single‐incision laparoscopic surgery has been reported to be useful and safe for other organs, such as in cholecystectomy.^15^, ^16^ In the area of colectomy, the usefulness and safety of SILC compared with multiport laparoscopic colectomy (MPLC) have been clarified. Five randomized control trials (RCTs) comparing SILC and MPLC in colectomy have been published, including our previous report.^10^, ^11^, ^12^, ^13^, ^14^ Those studies found no marked differences in the oncological or short‐term outcomes between SILC and MPLC. We also previously reported no significant difference in the oncological outcomes between SILC and MPLC in our RCT.^10^

In general, the advantages of SILC are considered to be a short wound length and improved cosmetic outcomes. However, the assessment of the cosmetic outcomes is a highly subjective evaluation. Maggiori et al^11^ reported that there were no significant differences in the postoperative pain or health‐related quality of life (HRQOL) at 6 months after surgery, although SILC had a significantly higher satisfaction with the scar aspect at 6 months after surgery. In contrast, in an RCT for SILC + 1, Wang et al^12^ reported that there were no significant differences in patient satisfaction with the scar aspect.

There have been only a few studies comparing HRQOL and patient satisfaction between SILC and MPLC for CRC patients, and there are some differences in results between previous RCTs. Therefore, the effect of SILC on HRQOL and patient satisfaction is still unclear. We conducted a RCT comparing SILC and MPLC in colectomy for colon cancer and previously reported the main results.^10^ Subsequently, we performed this examination to evaluate the benefit to HRQOL and patient satisfaction associated with these two approaches. This examination of subjective data was pre‐planned in the original study protocol, and the data were collected as part of that trial.

This sub‐study clarified the effect of SILC compared with MPLC for CRC on HRQOL and patient satisfaction, including wound pain and cosmetic outcomes.

## 2 METHODS

This was a pre‐planned sub‐study of an open‐label, multicenter, randomized, prospective trial comparing SILC and MPLC. We previously reported the trial design and main results of this RCT.^10^ A brief summary of this trial design is shown below. The study protocol was approved by the Ethical Advisory Committee of Yokohama City University School of Medicine and the institutional review board of each participating hospital before the study was initiated.

Between March 2012 and March 2015, a total of 200 patients with colon cancer located in the cecum, ascending colon, sigmoid colon or rectosigmoid with histologically proven adenocarcinoma or signet‐ring cell carcinoma stage 0‐III, according to the UICC TNM classification (7th edition), were recruited from three institutions of the Yokohama Clinical Oncology Group and randomly allocated to receive SILC or MPLC. This trial was registered with the Japanese Clinical Trials Registry as UMIN000007220, and all patients provided their written informed consent before enrolling in this trial. The inclusion criteria were an age of 20‐80 years, performance status ECOG 0‐1, no bulky tumor (≤4.0 cm in diameter), no history of gastrointestinal surgery except appendectomy, and no organ dysfunction or blood abnormality. The exclusion criteria were multiple cancers (disease‐free interval within 5 years), active infectious disease, severe comorbidities, medical history of mental and/or central nervous system disease, pregnant or lactating status, and preoperative treatment for colon cancer.

Randomization and data handling were performed by the Department of Biostatistics and Epidemiology Data Center of Yokohama City University. After confirming the inclusion/exclusion criteria and obtaining written informed consent, patients were randomized by means of computerized randomization following a fax to the data center on the morning of surgery. The allocated procedure was not concealed from the investigators or the patients. The sizes of the groups were balanced using the minimization method according to sex, age (<65 and ≥65 years old), and clinical stage (stage 0‐II, III).

### 2.1 Surgical procedure

All procedures were performed by five surgeons who had received endoscopic surgical skill accreditation for laparoscopic gastroenterological surgery from the Japan Society for Endoscopic Surgery.^17^ In both arms, complete mesocolic excision (CME) with central vascular ligation (CVL) was performed. Other details of the SILC and MPLC procedures have already been reported.^10^

### 2.2 Outcomes and statistical analyses

The main outcome in this sub‐study was the HRQOL score at 1 month after surgery. The primary outcome of this entire RCT was the incidence of postoperative complications within 30 days after surgery.

The sample size of this trial was determined as described below. A power analysis determined that 95 patients would be required to demonstrate a reduction in the incidence of postoperative complications within 30 days from 25% to 10% at a significance level of 5% and power of 80%. Subsequently, 100 patients were randomly assigned to each group.

The HRQOL score was measured using the Japanese version of the 36‐item Short Form Health Survey (SF‐36) version 2.0, which was based on the Japanese National Reference.^18^, ^19^ The questionnaires were mailed to the patients before and 1 month after surgery. The patients completed the questionnaires at home and then mailed it back to us.

The SF‐36 is a tool used worldwide for measuring HRQOL according to an inclusive standard and not a disease‐specific standard.^20^, ^21^, ^22^, ^23^ A genetic instrument was believed to be better suited to this sub‐study than a disease‐specific instrument because the assessment involved a wide range of disease processes. The SF‐36 was translated into Japanese and validated for the general Japanese population.^18^, ^19^ This tool contains 36 questions. The score is expressed numerically by the provided scoring algorithm. Eight different health‐related quality scales (eight subitems) comprising the physical function (PF), role physical (RP), bodily pain (BP), general health (GH), vitality (VT), social functioning (SF), role emotional (RE), and mental health (MH) were compared between the groups. The Physical, Mental, and Role/Social Component Summaries (PCS, MCS, RCS, respectively) that are calculated from these eight subitems were then compared in the Japanese version of the SF‐36. Suzukamo et al^24^ reported that the three‐component model is better than the two‐component model in Japan. Therefore, the three‐component model was also used in this sub‐study. The scores of each scale range from 0 to 100, with higher scores reflecting a better health status. The HRQOL survey using SF‐36 questionnaires was performed before surgery and 1 month after surgery. Regarding each item of the SF‐36, comparison between the SILC group and the MPLC group and comparison before and after surgery for each group were performed.

In addition, the rate of change in each item of SF‐36 before and after surgery was also examined. The proportion of patients with 10%, 15%, and 20% reduction of each score of the SF‐36 from baseline were compared between the two groups respectively.

The secondary outcome in this sub‐study was the patient satisfaction with the surgery and their postoperative clinical course. Seven questions were asked regarding patients' satisfaction at 1 month after surgery, based on the five‐case method. Patients who did not respond to all of the questions were excluded from this analysis.

We performed a post‐hoc subgroup analysis for HRQOL at 1 month after surgery, stratified by the postoperative complications (Clavien‐Dindo classification [CD] grade 0‐I vs ≥II).

All continuous data in this article are presented as the median (interquartile range [IQR]). All statistical analyses were performed with EZR (Saitama Medical Center, Jichi Medical University, Saitama, Japan^25^), which is a graphical user interface for R (The R Foundation for Statistical Computing). Differences between categorical and continuous variables were tested with Pearson's chi‐square test and the Mann‐Whitney *U* test, respectively. The results of the SF‐36 and the questions concerning the satisfaction of the patients were examined to determine whether or not they showed a normal distribution, but no obvious normality was observed. Therefore, non‐parametric tests were performed to analyze the results of this sub‐study. Differences between continuous variables were tested using the Mann‐Whitney *U* test when there was no correspondence between the two groups or Wilcoxon's signed rank test when correspondence was noted between the two groups. For the analysis of the questions concerning patient satisfaction by the five‐case method, we considered the five answer items as ordinal variables and used the Mann‐Whitney *U* test. *P*‐values <.05 were considered statistically significant.

## 3 RESULTS

Between March 2012 and March 2015, a total of 200 patients were included in this trial, 100 per arm. The clinicopathological characteristics and surgical outcomes of the 200 patients are presented in Table 1. The baseline factors were well‐balanced, and the surgical outcomes were similar between the MPLC and SILC arms. After excluding 18 patients (9.0%) who did not complete the SF‐36 questionnaire, 182 patients (91.0%) were ultimately included in the analysis of this sub‐study (92 patients in the SILC group and 90 patients in the MPLC group).

**TABLE 1:** Clinicopathological characteristics and surgical outcomes

Variable	SILC (n = 100)	MPLC (n = 100)	*P* value
Age (y)^a^	68 (61‐74)	67 (61‐74)	.671
Gender			
Male	56 (56.0)	56 (56.0)	1.000
Female	44 (44.0)	44 (44.0)	
BMI (kg/m^2^)^a^	22.9 (20.3‐25.2)	23.1 (21.1‐24.8)	.888
PS (ECOG)			
0	99 (99.0)	97 (97.0)	.621
1	1 (1.0)	3 (3.0)	
ASA physical status			
I	29 (29.0)	22 (22.0)	.330
II	71 (71.0)	78 (78.0)	
Tumor diameter (mm)^a^	26.5 (18.0‐40.0)	25.0 (15.0‐36.8)	.777
Location			
Cecum	16 (16.0)	9 (9.0)	.516
Ascending colon	21 (21.0)	21 (21.0)	
Sigmoid colon	49 (49.0)	54 (54.0)	
Rectosigmoid	14 (14.0)	16 (16.0)	
Operative procedure			
lleo‐caecal resection	17 (17.0)	10 (10.0)	.460
Right hemicolectomy	21 (21.0)	20 (20.0)	
Sigmoidectomy	42 (42.0)	51 (51.0)	
Anterior resection	20 (20.0)	19 (19.0)	
Operative time (min)^a^	150 (132‐174)	155.5 (136‐186)	.396
Blood loss (mL)^a^	5 (0‐15)	5 (0‐10)	.155
Conversion to open surgery	1 (1.0)	2 (2.0)	.561
Postoperative complication			
Total	12 (12.0)	15 (15.0)	.680
Grade III≥	8 (8.0)	5 (5.0)	.568
Reoperation	3 (3.0)	3 (3.0)	1.000
Pathological stage			
I	56 (56.0)	54 (54.0)	.105
II	14 (14.0)	25 (25.0)	
III	28 (28.0)	21 (21.0)	
IV	2 (2.0)	0 (0.0)	
Collected SF‐36 questionnaire	92 (92.0)	90 (90.0)	—
Collected questionnaire about patient satisfaction	92 (92.0)	89 (89.0)	—

Note

Values in parentheses are percentages, unless indicated otherwise.

Abbreviations: ASA, American Society of Anesthesiologists; BMI, body mass index; MPLC, multiport laparoscopic colectomy; PS, performance status (Eastern Cooperative Oncology Group); SILC, single‐incision laparoscopic colectomy.

^a^ Values are median (IQR: first quartile, third quartile).

**FIGURE 1:**
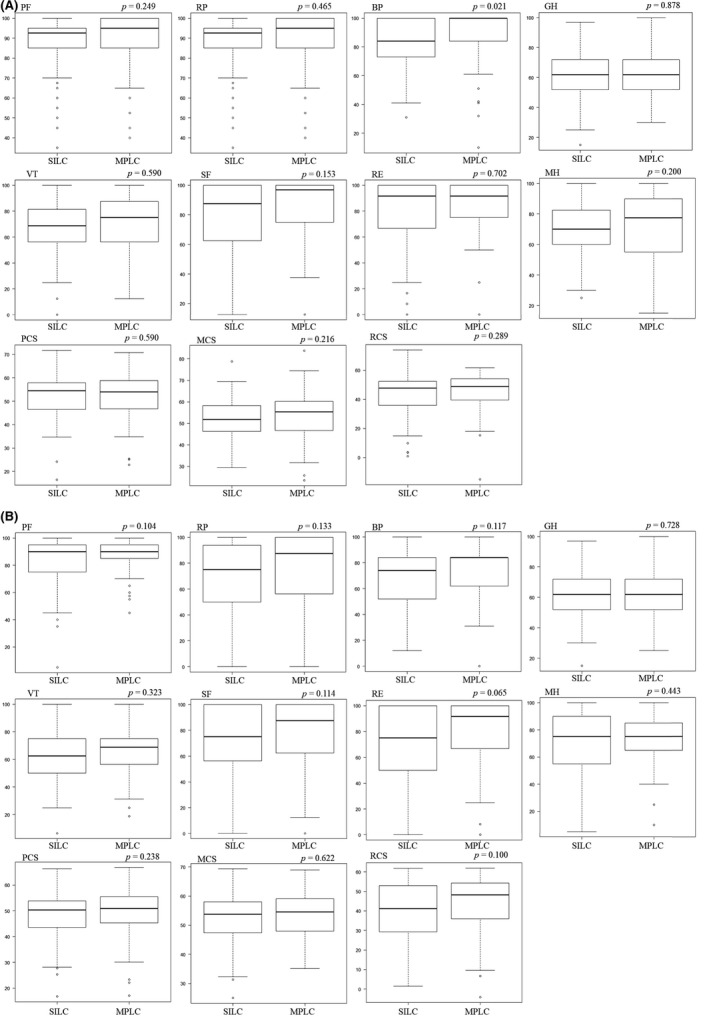
Health‐related quality of life (HRQOL) scores; (A) before surgery, (B) 1 mo after surgery. BP, bodily pain; GH, general health; MCS, mental component summary; MH, mental health; MPLC, multiport laparoscopic colectomy; PCS, physical component summary; PF, physical functioning; RCS, role/social component summary; RE, role emotional; RP, role physical; SF, social functioning; SILC, single‐incision laparoscopic colectomy; VT, vitality. All two‐group comparisons were obtained with Mann‐Whitney *U* test

The HRQOL scores before surgery (baseline) are presented in Figure 1A. There was no statistically significant difference between the two arms in all other subitems of SF‐36, except for the BP. The preoperative BP was significantly lower in the SILC group than the MPLC group.

The HRQOL scores at 1 month after surgery are shown in Figure 1B. None of the subitems or component summary scores of the SF‐36 differed significantly in the two arms. The changes in HRQOL before and after surgery are compared in the two arms respectively in Table 2. Among all subitems and component summary scores, the RP, BP, VT, and PCS were significantly lower at 1 month after surgery than before in both groups. The PF, GH, SF, MH, MCS, and RCS were not significantly different after surgery compared with before surgery in both groups. However, the RE was significantly lower after surgery than before only in the SILC group. Regarding the change rate of each item of SF‐36 before and after surgery, the proportion of patients with 10%, 15%, and 20% score reduction was compared respectively but no significant difference was observed between the two groups (the data were not shown).

**TABLE 2:** Comparison of changes in HRQOL before and after surgery in the two arms respectively

	SILC (n = 92)		*P* value^a^	MPLC (n = 90)		*P* value^a^
	Before surgery	After surgery		Before surgery	After surgery	
PF	92.5 (85.0, 95.0)	90.0 (75.0, 95.0)	.102	95.0 (85.0, 100)	90.0 (85.0, 95.0)	.123
RP	100 (75.0, 100)	75.0 (50.0, 93.8)	<.001	100 (75.0, 100)	87.5 (56.3, 100)	.003
BP	84.0 (73.5, 100)	74.0 (52.0, 84.0)	<.001	100 (84.0, 100)	84.0 (62.5, 84.0)	<.001
GH	62.0 (52.0, 72.0)	62.0 (52.0, 72.0)	.707	62.0 (52.0, 72.0)	62.0 (52.0, 72.0)	.962
VT	68.8 (56.3, 81.3)	62.5 (50.0, 75.0)	<.001	75.0 (56.3, 87.5)	68.8 (56.3, 75.0)	.008
SF	87.5 (62.5, 100)	75.0 (59.4, 100)	.167	96.9 (75.0, 100)	87.5 (65.6, 100)	.187
RE	91.7 (66.7, 100)	75.0 (50.0, 100)	.005	91.7 (75.0, 100)	91.7 (66.7, 100)	.398
MH	70.0 (60.0, 81.3)	75.0 (55.0, 90.0)	.174	77.5 (55.0, 90.0)	75.0 (65.0, 85.0)	.161
PCS	54.5 (46.5, 57.7)	50.1 (43.6, 57.7)	<.001	54.0 (46.8, 58.7)	51.0 (45.5, 55.5)	.001
MCS	51.9 (46.3, 58.2)	53.8 (47.4, 57.9)	.454	55.4 (47.1, 60.0)	54.5 (48.1, 59.1)	.992
RCS	47.8 (35.6, 52.4)	41.2 (29.4, 53.0)	.109	48.9 (39.6, 54.0)	48.3 (36.1, 54.3)	.216

Note

Values are median (IQR: first quartile, third quartile).

Abbreviations: BP, Bodily pain; GH, General health; HRQOL, health‐related quality of life; MCS, Mental component summary; MH, Mental health; MPLC, multiport laparoscopic colectomy; PCS, Physical component summary; PF, Physical functioning; RCS, Role/Social component summary; RE, Role emotional; RP, Role physical; SF, Social functioning; SILC, single‐incision laparoscopic colectomy; VT, Vitality.

^a^ Wilcoxon's signed rank test.

After excluding one patient who did not complete the questionnaire about patient satisfaction, 181 patients were included in the analysis of patient satisfaction (92 patients in the SILC group and 89 patients in the MPLC group). The patient satisfaction at 1 month after surgery is compared between the two arms in Table 3. There were no significant differences between the two arms in any of the seven questionnaire items concerning the surgery itself or the postoperative course.

**TABLE 3:** Comparison of patient satisfaction in SILC and MPLC at 1 mo after surgery

	SILC n = 92	MPLC n = 89	*P* value^a^
Q.1: Satisfaction for abdominal pain and discomfort, n (%)			
Extremely satisfied	11 (12.0)	11 (12.4)	.480
Satisfied	37 (40.2)	39 (43.8)	
Neither	32 (34.8)	31 (34.8)	
Dissatisfied	8 (8.7)	6 (6.7)	
Extremely dissatisfied	4 (4.3)	2 (2.2)	
Q.2: Satisfaction for the surgical wound pain and discomfort, n (%)			
Extremely satisfied	17 (18.5)	11 (12.4)	.742
Satisfied	37 (40.2)	42 (47.2)	
Neither	32 (34.8)	32 (36.0)	
Dissatisfied	5 (5.4)	2 (2.2)	
Extremely dissatisfied	1 (1.1)	2 (2.2)	
Q.3: Did the surgery differ from what you had in mind from the previous explanation?, n (%)			
Strongly disagree (as excepted)	24 (26.1)	14 (15.7)	.323
Disagree	41 (44.6)	47 (52.8)	
Neither	11 (12.0)	14 (15.7)	
Agree	11 (12.0)	10 (11.2)	
Strongly agree (totally unexcepted)	5 (5.4)	4 (4.5)	
Q.4: Did the surgery feel harder than what was explained in advance?, n (%)			
Strongly disagree (extremely well)	29 (31.5)	19 (21.3)	.447
Disagree	41 (44.6)	52 (58.4)	
Neither	16 (17.4)	10 (11.2)	
Agree	5 (5.4)	7 (7.9)	
Strongly agree (Not at all well)	1 (1.1)	1 (1.1)	
Q.5: Did your body recover smoothly after the operation and before you left the hospital?, n (%)			
Strongly agree (extremely well)	21 (22.8)	21 (23.6)	.260
Agree	49 (53.3)	55 (61.8)	
Neither	12 (13.0)	10 (11.2)	
Disagree	7 (7.6)	3 (3.4)	
Strongly disagree (Not at all well)	3 (3.3)	0 (0.0)	
Q.6: Did you feel inconvenienced in your life after the operation until you were discharged?, n (%)			
Strongly disagree (extremely well)	9 (9.8)	9 (10.1)	.985
Disagree	45 (48.9)	43 (48.3)	
Neither	21 (22.8)	20 (22.5)	
Agree	14 (15.2)	16 (18.0)	
Strongly agree (Not at all well)	3 (3.3)	1 (1.1)	
Q.7: Satisfaction with the entire surgery, n (%)			
Extremely satisfied	51 (55.4)	51 (57.3)	.741
Satisfied	35 (38.0)	34 (38.2)	
Neither	6 (6.5)	2 (2.2)	
Dissatisfied	0 (0.0)	1 (1.1)	
Extremely dissatisfied	0 (0.0)	1 (1.1)	

Note

Values in parentheses are percentages.

Abbreviations: MPLC, multiport laparoscopic colectomy; Q, question number; SILC, single‐incision laparoscopic colectomy.

^a^ Mann‐Whitney *U* test.

The result of the subgroup analysis for HRQOL at 1 month after surgery, stratified by the postoperative complications (CD grade 0‐I vs ≥II) were shown in Table 4. In this subgroup analysis, patients with postoperative complications (CD grade ≥II) had significantly lower PF, RP, VT, SF, RE, MH, and RCS than those with postoperative complications (CD grade 0‐I).

**TABLE 4:** Subgroup analysis of health‐related quality of life scores at 1 mo after surgery, stratified by postoperative complication severity

	CD Grade 0 ‐ I postoperative complications	CD Grade ≥ II postoperative complications	*P* value^a^
	n = 168	n = 14	
PF	90.0 (80.0, 95.0)	85.0 (48.8, 88.8)	.006
RP	87.5 (56.3, 100)	59.4 (39.1, 78.2)	.006
BP	74.0 (62.0, 84.0)	79.0 (31.3, 84.0)	.359
GH	62.0 (52.0, 72.0)	59.5 (46.3, 64.3)	.252
VT	68.8 (50.0, 75.0)	56.3 (32.9, 68.8)	.025
SF	87.5 (62.5, 100)	56.3 (28.1, 62.5)	.001
RE	91.7 (64.6, 100)	66.7 (33.3, 83.3)	.016
MH	80.0 (65.0, 90.0)	67.5 (45.0, 75.0)	.019
PCS	50.7 (44.5, 54.3)	48.9 (29.7, 56.7)	.502
MCS	54.4 (48.0, 58.8)	51.4 (45.6, 55.7)	.182
RCS	47.0 (34.1, 54.0)	28.7 (20.3, 39.2)	.002

Note

Values are median (IQR: first quartile, third quartile).

Abbreviations: BP, Bodily pain; CD, Clavien‐Dindo classification; GH, General health; MCS, Mental component summary; MH, Mental health; PCS, Physical component summary; PF, Physical functioning; RCS, Role/Social component summary; RE, Role emotional; RP, Role physical; SF, Social functioning; VT, Vitality.

^a^ Pearson's chi‐squared test.

## 4 DISCUSSION

In the present sub‐study, we compared HRQOL assessed by the Japanese version of the SF‐36 and the patient satisfaction between the SILC and MPLC among Japanese patients with cancer. There were no significant differences between SILC and MPLC for any of the subitems or component summary scores of the SF‐36, except for the preoperative BP in the SILC group compared with the MPLC group. In addition, no significant differences were found between the two arms in any of the questionnaire items regarding patient satisfaction at 1 month after surgery.

Single‐incision laparoscopic colectomy has the benefits of minimizing abdominal trauma from surgery for CRC. However, in a small pilot study that compared levels of biochemical markers between SILC and MPLC, the IL‐6, IL‐8, and CRP levels showed similar peaks and no significant differences.^26^ In several meta‐analyses comparing SILC and MPLC, SILC was suggested to be more beneficial in terms of blood loss, length of postoperative hospital stay, and wound length when performed by a skilled surgeon.^8^, ^9^ In several previous RCTs comparing SILC and MPLC, there were no significant differences in the short‐term results, except for a shorter combined scar length with SILC.^10^, ^11^, ^12^, ^13^, ^14^ Poon et al and Wang et al reported that the postoperative pain was significantly less in SILC, whereas Maggiori et al reported no significant differences in the postoperative pain.^11^, ^12^, ^13^ Wang et al also reported that the intraoperative blood loss was significantly less in SILC, whereas Huscher et al and Maggiori et al reported no significant differences in the intraoperative blood loss.^11^, ^12^, ^14^ In general, no significant differences have been reported in the short‐term results, including with regard to other oncological results. We also previously reported that SILC had no clear benefit over MPLC in our own RCT.^10^

Previous studies, including our own report, have mainly dealt with objective data, and only a few studies have compared subjective data, such as HRQOL and patient satisfaction, between SILC and MPLC. Maggiori et al^11^ reported that there were no significant differences in the postoperative pain or HRQOL at 6 months after surgery, but they did note a significantly higher satisfaction with the scar aspect at 6 months after surgery in the SILC group compared with the MPLC group. However, of the patients enrolled in their trial, only 28.8% had CRC, and many of the patients enrolled were surgical patients with benign disease, such as Crohn's disease, diverticulitis, and benign neoplasm.^11^ On the other hand, Wang et al^12^ reported no significant differences in HRQOL or patient satisfaction with the scar aspect. However, while all of the patients in that trial were CRC patients, they also all had a plus‐1 port and may not necessarily reflect the results that might be achieved with pure SILC compared to MPLC.^12^

Minimizing abdominal trauma may provide certain subjective benefits. However, the present sub‐study found no statistically significant difference in HRQOL at 1 month after surgery between the two groups. Although preoperative BP between both groups was significantly lower in SILC, there was also no significant difference in postoperative BP between SILC and MPLC groups. There was no significant difference between the two groups in the preoperative information collected according to the protocol of this trial, and it was unclear why preoperative BP was lower in the SILC group than in the MPLC group. However, we also examined the rate of change in BP before and after surgery, but no significant difference was observed between the two groups. On assessing the RE, the RE significantly decreased before and after surgery only in the SILC group (Table 1). Therefore, based on the present findings, SILC may be slightly inferior in terms of the RE than MPLC. In Japan, SILC is a technique that has been standardized, but it is not yet widespread and is not well known among the public population. Therefore, the satisfaction with conventional five‐port laparoscopic colectomy may potentially be compared to open surgery rather than SILC. The present study also suggested that MPLC had a sufficiently high degree of satisfaction as surgery for colon cancer. Of note, a "single incision" may not be as great a concern to colon cancer patients as we surgeons think. Immediately after surgery, patients' interest may be focused on whether the surgery was completed successfully and the cancer was treated. This vague anxiety about new procedures among the public, particularly a lack of surety concerning whether the cancer has been completely removed with a single incision, may be reflected in the significant reduction in RE after surgery in the SILC group.

In the result of this study, there was no significant difference in patient satisfaction, despite a significant difference in RE between the two groups. The RE scores of the SF‐36 are low when there is a psychological problem when working or doing daily activities in the past 1 month. In our questionnaire of patient satisfaction, Q6 was the only item potentially associated with RE in SF‐36. In addition, Q6 is also an item that asks about the inconvenience of daily life until discharge. During hospitalization, there were not many such scenes in terms of work and daily activities, and it was considered that there are few RE‐related situations. Since patients were discharged about a week after surgery, RE on activity over the past 1 month could be more strongly influenced by the 3 weeks spent at home. In addition, Q3 and Q4 seemed to be more closely related to MH than RE. These were considered to be the factors that did not make a difference in patient satisfaction despite a significant difference in RE only in the SILC group.

The post‐hoc subgroup analysis revealed that postoperative complications (CD grade ≥II) influenced HRQOL at 1 month after surgery. Postoperative complications (CD grade ≥II) were associated with decreased PF, RP, VT, SF, RE, MH, and RCS. It became clear that the occurrence of severe postoperative complications had not only an objective index such as extension of hospital stay, but also adverse effects on physical activity, mental aspects, and social aspects.

Several limitations associated with the present study warrant mention. First, the sample size of this sub‐study was determined based on the primary outcomes and might not be relevant for the power of this sub‐study. Second, the SF‐36 and questionnaire concerning patient satisfaction were obtained only 1 month after surgery. So soon after surgery, patients are largely interested in whether the cancer has been cured, so interest in the wound may still be low at this point. From a long‐term perspective, satisfaction with the wound may change over time, e.g. 1 year after surgery. Third, the patients were not blinded, which might have influenced the outcomes. However, the authors believe that the findings of this study will provide a firm foundation for future studies.

In conclusion, SILC was similar to MPLC in terms of HRQOL and satisfaction among patients undergoing laparoscopic colectomy for cancer. However, SILC may be inferior in terms of RE.

## DISCLOSURE

Funding: This study received no specific grant from any funding agency in the public, commercial, or not‐for‐profit sectors.

Conflict of Interest: The authors declare that they have no conflicts of interest.

Author contribution: The contribution of each author to this reported work as follows. Hiroki Ohya and Jun Watanabe: conception and design, acquisition of data, analysis and interpretation of data and drafting the article and final approval of the version to be published. Yusuke Suwa, Hirokazu Suwa, Mayumi Ozawa, Atsushi Ishibe, Shoichi Fujii, Kazumi Kubota, Chikara Kunisaki, and Itaru Endo: conception and design, acquisition of data and revising the article critically for important intellectual content and final approval of the version to be published.
